# Analysis of the Mechanical Properties of Structural Ceramics Made from Aggregate Washing Sludge and Manganese Mining Waste

**DOI:** 10.3390/ma17174427

**Published:** 2024-09-09

**Authors:** Juan María Terrones-Saeta, Vanesa Domínguez, Daniel Ramos, Emilio Romero, Juan Asensio-Lozano

**Affiliations:** 1 Department of Industrial Engineering and Civil Engineering, Area of Mechanics of Continuous Media and Theory of Structures, Higher Technical School of Engineering of Algeciras, University of Cádiz, 11002 Cádiz, Spain; 2 Department of Mining, Mechanical, Energy and Construction Engineering, Higher School of Engineering, University of Huelva, 21007 Huelva, Spain; vanesa.dominguez@dimme.uhu.es (V.D.); daniel.ramos@dimme.uhu.es (D.R.); romaci@uhu.es (E.R.); 3 Department of Materials Science and Metallurgical Engineering, University of Oviedo, 33004 Oviedo, Spain; jasensio@uniovi.es

**Keywords:** mining waste, circular economy, structures, mechanical properties, modelling, sustainability

## Abstract

The construction sector is presently among the most resource-intensive industries, driving a substantial body of research dedicated to the development of more sustainable materials to address these demands. A particularly promising approach within the framework of the circular economy is the repurposing of waste as a principal raw material for the creation of new construction products. Within this context, the primary aim of this study is to engineer ceramic materials for brick production using 100% waste-derived inputs, specifically aggregate washing sludge and manganese mining by-products. To evaluate the potential of these sustainable ceramic materials, an extensive investigation was conducted, encompassing both physical and mechanical testing, as well as a thorough characterisation of the waste inputs. For this purpose, a series of ceramic specimens were fabricated with varying proportions of mining residues and aggregate washing sludge, adhering to the conventional protocols employed in the manufacture of ceramic bricks. The results demonstrate that these sustainable ceramics exhibit a linear shrinkage reduction of up to 5% compared to traditional clay-based ceramics. Furthermore, they show water absorption levels—whether via capillarity, cold water, or hot water absorption—that are up to twice those observed in conventional clay ceramics, while maintaining comparable density values. This increased absorption, however, correlates with a reduction in mechanical strength at higher concentrations of manganese waste, yet the material continues to meet the minimum strength requirements as specified by industry standards for such products. In conclusion, this research introduces a novel, sustainable ceramic material that not only reduces economic and environmental costs but also adheres to the required performance criteria for construction applications.

## 1. Introduction

Mining is a particular phenomenon in terms of waste generation [1]. This practice, whose origins in the Iberian Peninsula are lost in antiquity, ranges from the exploitation of polymetallic sulphides at a depth of 300 m to the extraction of aggregates in gravel pits near rivers [2].

In general, the extraction of the marketable product requires the extraction of significant amounts of waste, which for economic reasons is disposed of in the vicinity of the extraction site [3]. In addition, in order to achieve the purity or concentration required to market the desired product, the extracted mineral mass must undergo various processes that generate residues [4].

The search for more environmentally friendly solutions and the promotion of sustainability are current trends [5]. This shift in focus is mainly due to the growing environmental awareness of the population, the increasing scarcity of resources and, as a result, the implementation of stricter environmental regulations and standards in various countries [6].

New sustainable materials are being developed in the construction sector to reduce environmental impact and greenhouse gas emissions [7]. This sector is known for its high pollution levels due to the consumption of large quantities of raw materials, poorly optimised industrial processes, and the manufacture of huge quantities of products [8]. All of these factors directly contribute to the future scarcity of essential raw materials and high energy consumption [9].

In the field of construction, particularly building, a wide range of materials are used. Among these, ceramics, including bricks, are one of the most commonly used elements due to their excellent mechanical performance, cost-effectiveness, and durability. These ceramic products have a long history in construction. Although these bricks have advantages, their manufacture involves costly operations to extract virgin materials, such as clay, which generates CO_2_ emissions [10]. Additionally, it requires a sintering process at high temperatures, approximately 950 °C. This scenario highlights the need to transform this product into more sustainable alternatives, a topic that has been the subject of several research studies in search of environmentally friendly solutions [11].

To achieve a more sustainable ceramic product, the formulation and manufacturing process of bricks have undergone several modifications. China, a major producer, has limited its production to prevent the loss of arable land [12]. However, slowing down the construction sector, which provides numerous benefits to the population, may not be the optimal solution.

On the other hand, sustainable materials like geopolymers have been developed [13]. They use waste in their composition and have less polluting manufacturing processes. However, this is a line of study that requires further development, and durability issues need to be addressed. Additionally, incorporating manganese waste has been explored as an option for manufacturing ceramic bricks, making the process more economical and environmentally sustainable [14].

The use of waste in brick making has resulted in numerous success stories, thanks to a variety of waste materials [11,15,16]. This technique significantly reduces the need to use virgin materials such as clay, thereby reducing extraction operations, associated emissions, and environmental impact in the region [17]. On the other hand, the integration of waste into ceramic materials can result in a reduction in the economic cost of the final product and a decrease in the disposal of unused waste [18].

This approach offers economic and environmental benefits, as well as the potential to impart specific properties to the final product, such as improved thermal and acoustic insulation and increased lightness of the material. It is important to note that this incorporation can be achieved without significant changes to the production industry’s processes or machinery [19].

Recycling is an option that offers economic and environmental benefits. It reduces the amount of waste deposited in landfills and gives new life to materials, making it fully compatible with the principles of the circular economy [20]. Furthermore, some waste materials can be harmful to the environment due to their chemical composition, potentially contaminating surface or groundwater and affecting vegetation and fauna. These wastes should be handled with special care and integrated into materials that retain the conflicting elements or chemical compounds effectively. Incorporating the polluting wastes into ceramic materials is one of the best options, as the ceramic matrix retains these pollutants in high proportion and prevents their leaching [21].

In particular, during the production of aggregates for the construction sector, considerable waste is generated, which can sometimes have a significant environmental impact. Aggregate production involves three main stages: crushing of the material, particle size grading and, in most cases, washing of the final product.

After completing the crushing and grading stages, the aggregates are washed to remove any ultra-fine material. The liquid phase is then separated from the solid phase in several stages until the final sludge is obtained [22].

In the construction sector, the European industry produces around 5 million tonnes of sludge annually through the cleaning of aggregates for further treatment and sale. This sludge is usually deposited in large ponds for containment but is currently of no use [23].

In addition, manganese waste from former mining dumps in the Iberian Pyrite Belt can contaminate surface and groundwater due to its chemical composition. In addition, their small particle size can have a detrimental impact on vegetation [14].

Based on the above, and with the aim of developing a new circular economy, this study aims to provide an ecological solution that reduces the accumulation of waste and promotes the development of sustainable materials that can be industrialised. Therefore, this research explores the integration of manganese waste in aggregate washing sludge for the production of ceramic bricks.

To achieve this objective, the analysis of the sludge and manganese wastes begins with an examination of their physical and chemical properties. Subsequently, several groups of samples were made using different combinations of sludge and manganese residues, from 100% sludge to 100% manganese residues. The samples were subjected to sintering and then the quality of the material was evaluated using various physical tests for ceramics. The objective of these tests was to analyse the variation in the properties of the samples with different percentages of Mn residues and sludge compared to traditional clay. Finally, the compressive strength test revealed the ideal combinations of sludge and manganese residues that provided ceramic materials with properties suitable for commercialization.

The results of the various tests showed that it is feasible to use both wastes, aggregate washing sludge and manganese waste, for the production of ceramic materials for the construction sector.

## 2. Materials and Methods

This section describes the materials used in this research, both wastes, and the methodology followed to prove that it is feasible to manufacture ceramic materials for the construction sector with the use of the detailed by-products as raw materials.

### 2.1. Materials

The materials mainly used in this research are sludge generated in aggregate washing plants and manganese waste from the tailings of former manganese mines. Both materials are described below.

#### 2.1.1. Sludge from Aggregate Washing

**Figure 1:**
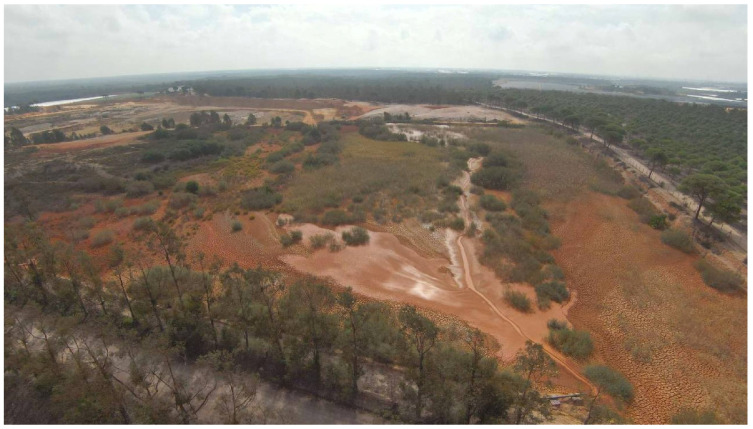
Image of the ponds in which sludge from aggregate washing is deposited.

The sludge used for the development of this research comes from the gravel pit located in the south of Spain, more specifically in Huelva, Figure 1. This company has a surface area of 311 hectares, with 285 hectares authorised as an exploitation area. In the quarry, natural gravel is extracted, which is subjected to a washing and subsequent classification process, with the aim of obtaining a product with certain characteristics. It is during this washing process that sludge is produced, which is transported by pipes from the washing area to ponds where it accumulates.

Taking into account that the daily production of an average-sized aggregate washing plant is approximately 700 tonnes/day, a total of 112 tonnes of sludge is generated per day, which is around 16% of the total production mentioned.

Although it is true that this sludge does not contain hazardous substances that could damage or contaminate the soil, it does have other environmental disadvantages, particularly the large surface area required for the storage and containment of this sludge. In addition to the farm from which the samples were taken for this study, the province of Huelva has more than ten other farms that generate this same type of waste, resulting in a high accumulation of these materials and the corresponding surface area contamination.

Different samples of this sludge were taken, with very similar physicochemical characteristics. Once these samples had been taken, they were dried in an oven at 105 °C, and then the material was crushed. In this way, a reduced particle size of the material was obtained, avoiding conglomerations. This aggregate washing sludge has a particle size of less than 250 μm, as could be demonstrated in the laboratory.

#### 2.1.2. Manganese Mining Wastes

In the 19th century, manganese mining activity left a significant mark in Spain, with the province of Huelva standing out as a leader in the production of this mineral. This region is home to most of the mines in operation dedicated to the extraction of manganese, and associated with it we find a large area affected by mining dumps, mainly composed of chert gangue.

At present, Huelva has an inventory of at least 159 abandoned manganese mines, many of which show abandoned dumps, contributing to the degradation of the landscape and affecting the environmental conditions of the environments where they are located.

Chert gangue, which we found in the dumps of former manganese mines, is a compound with an average SiO_2_ content of more than 80%. Minimal amounts of sodium, calcium, phosphorus, aluminium, potassium, magnesium, or compounds of these minerals are found in them, which means that chert gangue is considered to be sterile material. It is this characteristic that allows it to be used in the manufacture of other materials.

The samples taken were obtained from different abandoned mines in the province of Huelva, the most significant being the Santiago and Soloviejo mines.

After carrying out the sampling campaign for subsequent use in the creation of specimens, a series of processes were carried out to reduce the particle size to the desired size. Firstly, the samples were introduced into a jaw crusher. The resulting material was then passed through a cylinder crusher. After that, a particle mill was used to further reduce the particle size. Lastly, a particle size analysis was carried out to obtain the particle size necessary for the production of the different test specimens. Finally, and after carrying out the whole process described above, a particle size of less than 250 μm was achieved for the manganese residue.

### 2.2. Methodology

The method followed in this project is based on a series of tests carried out in a specific order, so that clear and concise results are obtained from each one of them on the viability of incorporating manganese waste into ceramic materials.

Firstly, following a rigorous scientific and objective scheme, the materials used in the manufacture of the ceramic pieces are analysed to corroborate that they are suitable for this process, as well as the compatibility between both materials. To this end, different physical and chemical tests are carried out on the materials used. 

Once all the specimens were formed and sintered in a muffle furnace at a temperature of 950 °C after prior drying in an oven, the different physical tests were carried out to provide the necessary information on the resulting material in relation to the percentage of residue in each sample. 

Once the physical properties of the different families of specimens have been evaluated, the mechanical compression test is carried out in accordance with the regulations in force, which will provide us with information on the maximum resistance that each specimen can withstand.

It is worth noting that all tests and experiments have been conducted in a laboratory with strictly controlled environmental conditions. The laboratory temperature has been maintained at 20 °C, with a variation of ±3 °C, while the relative humidity has ranged between 33% and 55%. These controlled conditions have been essential to ensure the accuracy and reproducibility of the results, minimising the impact of external factors that could affect the integrity of the tests and experiments performed.

#### 2.2.1. Physicochemical Characterisation of the Materials

Following a rigorous scientific and objective scheme, the materials used in the manufacture of the ceramic pieces are analysed to corroborate that they are suitable for this process. To this end, this section describes the different physical and chemical tests carried out on the materials used.

The physical tests carried out on these materials were the calculations of the real particle density (according to standard UNE-EN 1097-7), which is of vital importance for the characterisation of a clay. At the same time, chemical tests were carried out to determine the composition of the products used: Elemental Analysis (LECO, St. Joseph, MI, USA), loss on ignition, and X-ray Fluorescence (Thermo Fisher Scientific, Waltham, MA, USA). In order to mix both materials adequately, it was necessary to have a particle size of less than 0.25 mm, so the sludge had to be subjected to a grinding process and subsequent sieving until the desired grain size was achieved.

#### 2.2.2. Forming of Specimens: Physical and Mechanical Tests and Colourimetric Tests on the Families of Specimens

Once the analyses described above have been carried out on the materials to be used, the different families of test specimens are prepared. 

Firstly, a family of specimens made up of 100% clay is made, which will serve as a reference to compare the results obtained after the different tests to the other families. At the same time, families of specimens are made with sludge and waste from waste dumps in different percentages of combination of both materials. These percentages vary from 100% sludge to 100% landfill waste, with growth rates of 25%. Table 1 shows the different families of ceramic specimens formed with varying percentages of Mn waste.

To form each family of specimens, firstly, the sludge and the manganese residues are mixed in the percentage corresponding to each one, and once both materials are mixed dry, 10% water is added to the mixture, which is mixed again until a homogeneous mass is obtained. This material is then poured into the matrix and compacted as indicated below. 

Each family consists of four specimens, made by compression in a matrix. The die has an inner dimension of 40 mm × 20 mm, where the material is poured and then compacted by applying a load of 20 MPa for 1 min. Once the mixture is obtained, 20 g of the mixture are used to make each test specimen.

Once the specimens have been formed, they are removed from the matrix, and measurements are taken of their length, width, and height. Subsequently, they are placed in an oven to dry at a temperature of 105 °C for 24 h. After this time, the specimens are removed from the oven and, once they are at room temperature, the geometric measurements are taken again and the dry mass of each specimen is taken.

The next step would be to sinter the materials. To accomplish this, the specimens are placed in an oven at a temperature of 950 °C, maintaining this temperature for 1 h. The samples are then allowed to cool, and the mass and dimensions are taken again. In this way, the linear shrinkage and weight loss of the specimens is determined (UNE-EN 772-16 standard). 

The next tests to be carried out are the capillary water absorption (UNE-EN 772-11 standard), cold water absorption (UNE-EN 772-21 standard), and boiling water absorption tests. In turn, after subjecting the test specimens to the boiling water process, the submerged mass and the mass with dry saturated surface of the different test specimens are obtained in order to calculate the open porosity and density of the ceramic elements produced (UNE-EN 772-4 standard).

Finally, the colour of the different specimens was measured by means of a colorimeter (RGB-2, PCE, Meschede, Germany). In this way, the colour of the samples and the influence of the manganese residue on this property could be analysed objectively.

#### 2.2.3. Mechanical Testing

Once all the aforementioned tests have been carried out, the compression test is performed in order to determine the strength of the specimens (UNE-EN 772-1 standard). 

In this way, the mechanical suitability of the material for use as brick ceramics can be objectively assessed.

#### 2.2.4. Discussion of the Results Obtained in Comparison with Previous Research

In this research, following the evaluation and discussion of the experimental results, a final discussion has been conducted in which the advantages of the developed sustainable ceramics are compared with those from previous studies. This comparative analysis focuses on highlighting the specific benefits of the new sustainable ceramics in relation to the materials examined in earlier research. The discussion addresses key aspects such as the stability of physical and chemical properties, durability, reduction of environmental impacts, and waste integration. This comparative approach not only provides a detailed insight into the advancements achieved but also allows for a critical assessment of how the current sustainable ceramics surpass or maintain the standards set by traditional ceramics and previous developments in the field.

## 3. Results

This section describes the results of the tests detailed in the methodology, as well as the partial conclusions that can be drawn from them. These partial conclusions allow us to corroborate the final objective of this research, which is the development of ceramic materials to replace traditional ceramics with mining waste.

### 3.1. Physicochemical Characterisation of the Materials

The results obtained from the tests carried out in relation to the physicochemical characterisation of the materials used in this project are detailed below.

- The Real Density obtained for the sludge was 2.67 Tn/m^3^, while for the manganese waste, a Real Density of 3.07 Tn/m^3^ was obtained. The difference in density between the two wastes is not very high, however, it must be checked during the manufacturing process that the homogenisation of the two materials is correct.
- The results obtained in the elemental analysis test are shown in Table 2.

**Table 2:** Results of the elemental analysis test for the determination of carbon, nitrogen and hydrogen in samples of aggregate washing sludge and manganese residues.

	% N	% C	% H
Sludge	0.034 ± 0.002	0.312 ± 0.002	0.012 ± 0.001
Mn waste	0.151 ± 0.001	2.244 ± 0.042	0.074 ± 0.004

As can be seen in the table above, the percentage of the most volatile chemical elements is very low. Therefore, it can be determined that both materials are suitable for use in ceramics, as the percentage of organic matter is very low.

- The results obtained for ignition loss are shown in Table 3.

**Table 3:** Loss on ignition of aggregate washing sludge and manganese waste.

Sample	% LOI
Sludge	1.92 ± 0.012
Mn waste	5.11 ± 0.056

The loss on ignition of both wastes is low, reflecting that the existence of volatile chemical elements, typical of organic matter, is very low.

- X-ray fluorescence test.

The results of the X-ray fluorescence tests (Table 4) show that the aggregate wash sludge has a composition very similar to that of a common clay. The percentage of silicon and aluminium is predominant, combined with lower percentages of sodium, potassium and iron. The neutrality of this material is noteworthy, as there are no contaminants or volatiles that could impair the quality of the ceramic material.

Moreover, the X-ray fluorescence test of the manganese residues shows a high percentage of manganese, which is to be expected considering their origin. In addition, due to the geology of the area in which these wastes are found, there are high percentages of silicon and iron. To a lesser extent, chemical elements such as aluminium and calcium can be found. Other elements harmful to the environment such as zinc, barium, lead, or arsenic are not present.

### 3.2. Physical Tests on the Families of Specimens

The results obtained in the different physical and mechanical tests carried out on the different families of specimens, as well as the conclusions that can be drawn from them, are set out below.

**Figure 2:**
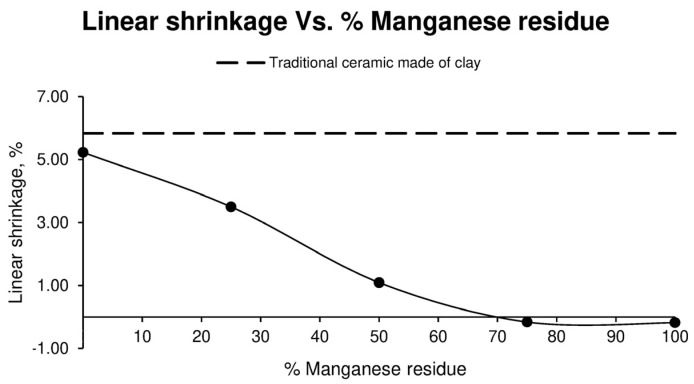
Linear shrinkage of different ceramic specimen families formed with aggregate washing sludges and increasing percentages of manganese mining residues, compared to the values of ceramics formed with clay.

Accordingly, the linear shrinkage test of the different families of conforming ceramics are shown in Figure 2.

After observing the results obtained regarding the linear shrinkage of the different families of specimens, it can be seen that the higher the amount of Mn residue added to the sample, the greater the linear shrinkage. When 75% Mn residue and upwards is reached, it can be observed that no linear shrinkage occurs, but, on the contrary, a slight expansion occurs. 

Linear shrinkage is a parameter of vital importance when forming parts with characteristic dimensions, since the possible decrease in size that the part may undergo depending on the percentage of Mn residue it contains must be taken into consideration. This decrease in linear shrinkage is directly related to the particle size of the manganese residue and, in turn, to its plasticity, which is significantly lower compared to that of clay.

**Figure 3:**
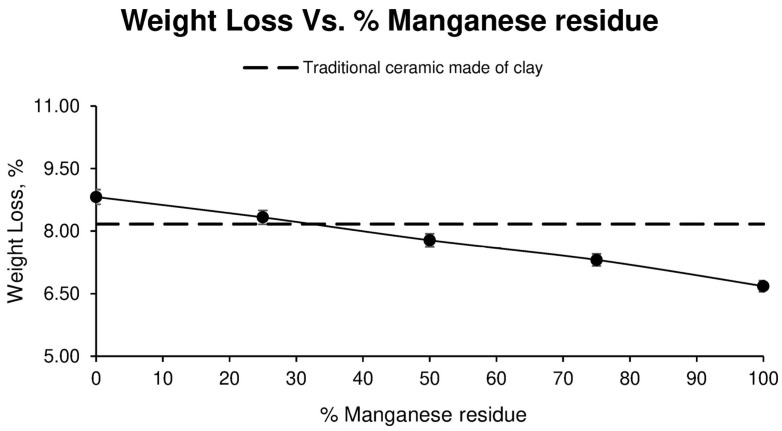
Weight Loss Linear of different ceramic specimen families formed with aggregate washing sludges and increasing percentages of manganese mining residues, compared to the values of ceramics formed with clay.

Regarding weight loss, as shown in Figure 3, in the first two families of specimens, those corresponding to 0% and 25% Mn residue, a higher mass loss is observed than that produced in the clays (which are used as a reference to analyse the results obtained in the different families of specimens), whereas, in the following families of specimens, a lower mass loss is observed as the amount of Mn residue increases. However, the results depend directly on the loss of ignition of both residues and are very similar to those of the clay.

**Figure 4:**
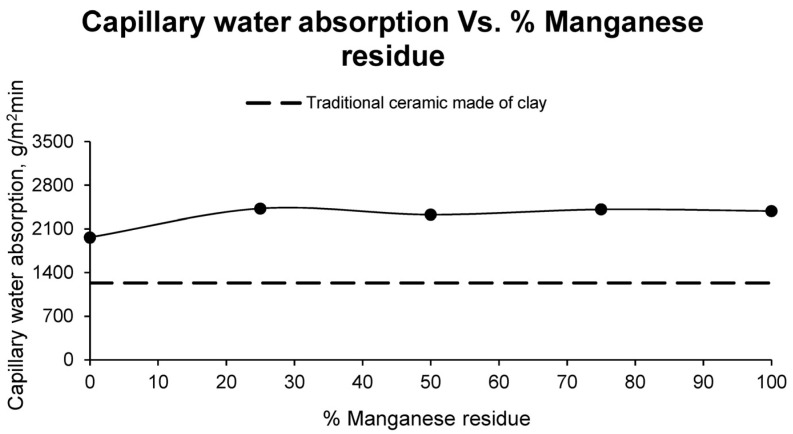
Capillary water absorption of different ceramic specimen families formed with aggregate washing sludges and increasing percentages of manganese mining residues, compared to the values of ceramics formed with clay.

The results of capillary water absorption of the different families of ceramics as a function of the percentage of manganese mining residue are shown in Figure 4.

Water absorption is a very important quality in ceramic materials, since it directly affects the useful life of the brick and indirectly affects the open porosity. The higher the suction, the higher the porosity and, consequently, the lower the strength of the piece. 

When analysing the results obtained, it is observed that the absorption increases with the addition of Mn residue to the sample, varying from 1962.93 g/m^2^min as an average value for the family of specimens made up entirely of sludge to values ranging between 2427.79 g/m^2^min and 2327.94 g/m^2^min. It should be noted that the results obtained for the test specimens exceed those obtained for the clays, whose absorption is 1230.77 g/m^2^min. It can be observed that the results are very similar to each other, although they are superior to those obtained for the clay ceramics.

**Figure 5:**
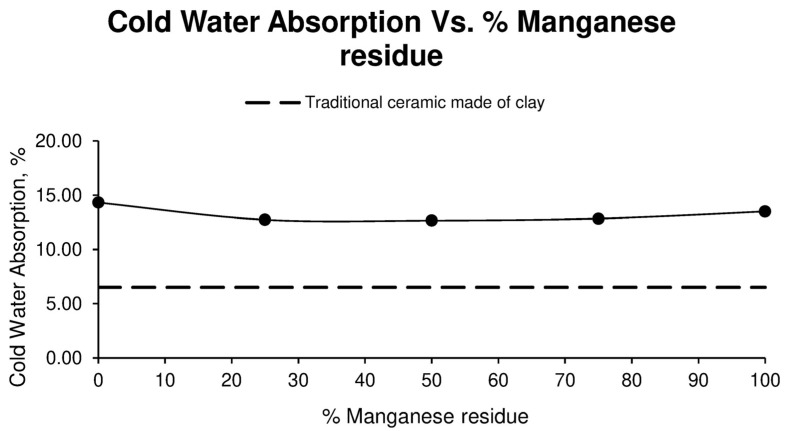
Cold water absorption of different ceramic specimen families formed with aggregate washing sludges and increasing percentages of manganese mining residues, compared to the values of ceramics formed with clay.

On the other hand, the cold water absorption of the ceramics as a function of the percentage of manganese residue is shown in Figure 5.

After analysing the results obtained, it can be seen that in the families of specimens in which sludge and Mn residues are used, the value obtained is very similar, between 12.66% and 12.84%, while for the families of specimens formed using only one material, the results are somewhat higher. In the case of the family made up of 100% sludge, 14.32% cold water absorption was obtained, while in the family made up entirely of Mn waste, 13.50% absorption was obtained. It should be noted that all these values are significantly higher than the value obtained for clays.

**Figure 6:**
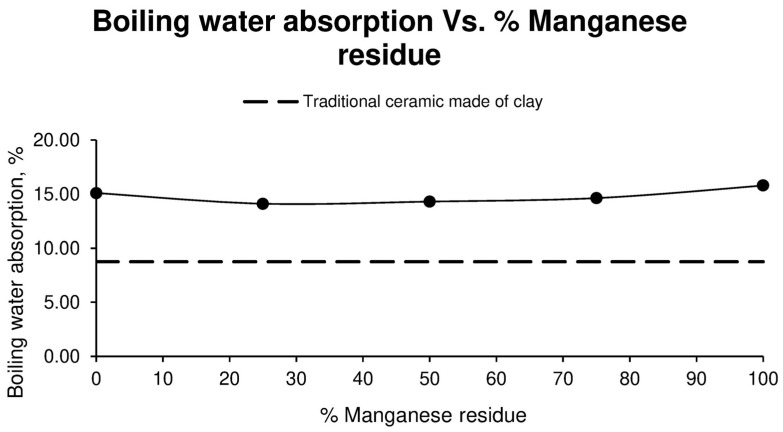
Boiling water absorption of different ceramic specimen families formed with aggregate washing sludges and increasing percentages of manganese mining residues, compared to the values of ceramics formed with clay.

Correspondingly, the results of the boiling water absorption of ceramics made with aggregate washing sludge and manganese residues are shown in Figure 6.

As can be seen above, the value obtained from the specimens made up of 100% sludge is 15.10%, whereas, when Mn residue is added, the value decreases with respect to that of the first family and, as the percentage of Mn residue increases, the absorption of boiling water increases.

The results obtained for capillary water absorption, cold water absorption, and boiling water absorption clearly indicate that ceramics formed with aggregate washing sludge develop a material with a higher number of interconnected pores, which facilitates greater water absorption. Consequently, it is understood that ceramics made with sludge have a more open structure compared to traditional ceramics. However, it is noteworthy that the addition of manganese mining residue does not adversely affect the values initially observed for the specimens composed solely of aggregate washing sludge; instead, the values remain virtually constant. Therefore, all evaluated combinations of both residues would meet the established standards.

Below shows the results obtained in relation to the bulk density of the pieces tested.

**Figure 7:**
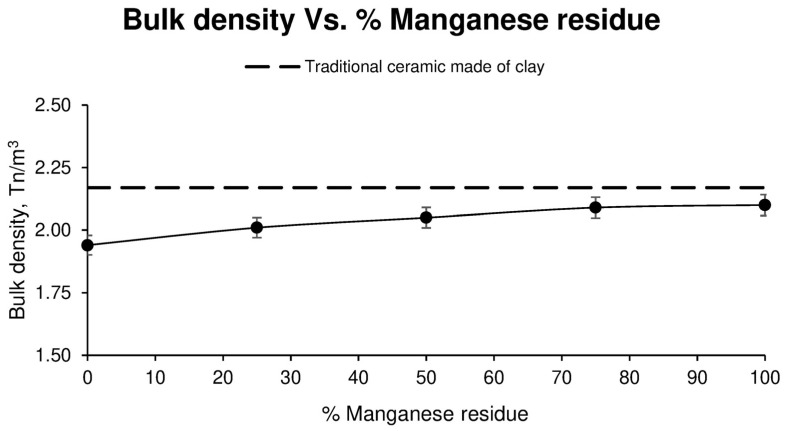
Bulk density of different ceramic specimen families formed with aggregate washing sludges and increasing percentages of manganese mining residues, compared to the values of ceramics formed with clay.

As can be seen in Figure 7, the bulk density varies according to the amount of Mn residue in the sample; the higher the amount of residue, the higher the bulk density. This is due to the fact that the density of the manganese residue is higher than that of the sludge used.

The results obtained in the colourimetry test are shown in Table 5 below.

**Figure 8:**
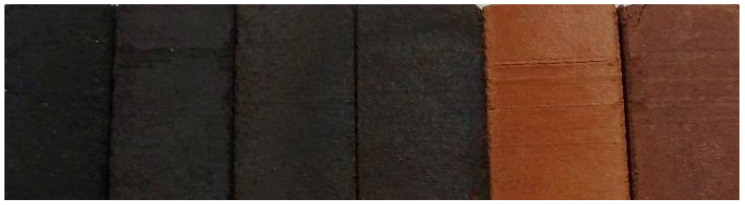
Final appearance of the different families of specimens.

The following illustration (Figure 8) shows the different shades presented by each family of specimens. The one on the left is composed of 100% Mn residue, followed by the family formed by 75% residue and 25% sludge. The third one on the left is formed by 50% residue and 50% sludge, the fourth one by 25% residue and 75% sludge, the next one is formed by 100% sludge, and the specimen on the right is the one used as reference to analyse the results and is formed by 100% clays. 

### 3.3. Determination of the Compressive Strength

**Figure 9:**
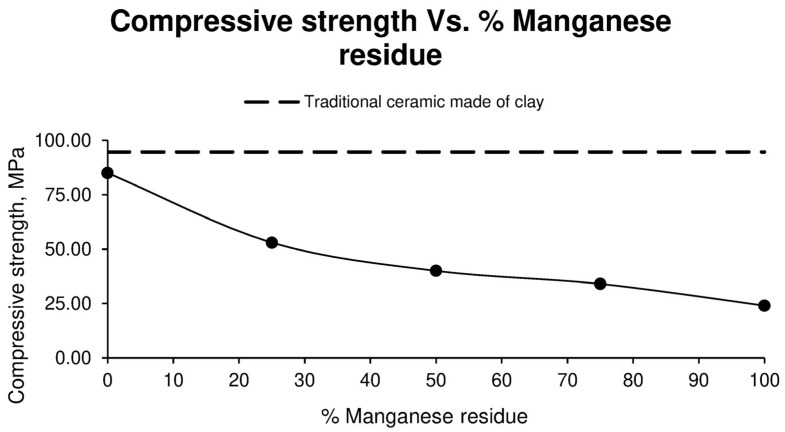
Compressive strength of different ceramic specimen families formed with aggregate washing sludges and increasing percentages of manganese mining residues, compared to the values of ceramics formed with clay.

For a better visualization of the results obtained from the compressive strength test of the different specimens, Figure 9 is shown below. 

As can be seen in the previous Figure, the higher the percentage of manganese residue in the sample, the lower the compressive strength, with the family of specimens made with 0% residue offering the best results with respect to those obtained from the specimens made entirely with clays.

Therefore, it can be asserted that ceramics manufactured with aggregate washing sludges exhibit strengths very similar to those of traditional ceramics, suggesting that this type of residue could be used as a substitute for clay in conventional ceramic industries. On the other hand, manganese residue adversely affects the material’s strength, which is expected given its origin and chemical composition. However, it is suggested that employing higher temperatures during the sintering process could alleviate this issue and enhance the mechanical properties of the final product.

### 3.4. Discussion of the Results Obtained in Comparison with Previous Research

The incorporation of aggregate washing sludge and manganese mining waste in the manufacture of ceramic bricks offers several notable advantages compared to other types of ceramics that use various types of waste. Firstly, ceramics produced with these wastes exhibit remarkable stability both over time and across different batches, maintaining consistency in their physical and chemical properties. Unlike sewage sludge or biomass ash, for instance, whose physical and chemical properties can vary depending on seasonal periods or the type of biomass used [24,25], aggregate washing sludge and manganese waste ensure a more homogeneous product [26].

Furthermore, ceramics manufactured with aggregate washing sludge and manganese mining waste display acceptable physical and mechanical properties across all combinations of these materials. In contrast, other wastes may compromise the feasibility of their incorporation in large percentages due to reduced strength and durability of the final product [27,28].

A key advantage is that the wastes under study do not possess contaminating characteristics, thereby minimising the risk of harmful leachate formation. This aspect is crucial compared to other ceramic materials that may release hazardous contaminants [29,30].

Additionally, the use of 100% of these wastes in the ceramic composition represents a significant advantage, as it allows for complete integration without relying on reduced percentages of waste, unlike other types of ceramics that incorporate waste only in small proportions [31]. This approach not only optimises waste utilisation but also provides a more economical and ecological solution.

Finally, the manufacturing process for these ceramics with wastes requires minimal modifications to traditional ceramic industry practices. Existing equipment can be used with few adaptations, unlike other methods that necessitate significant changes in infrastructure [32]. This facilitates the adoption of sustainable practices in the industry without the need for substantial additional investments.

This research opens several avenues for future investigation. Long-term ageing tests are recommended to assess the durability of the ceramics over time. Additionally, studying the materials’ exposure to adverse conditions could provide insights into their performance under extreme conditions. Exploring variations in sintering temperatures may also enhance the final product’s properties. These studies will contribute to optimising the use and integration of sustainable ceramics in industry.

In conclusion, ceramics developed from aggregate washing sludge and manganese mining waste not only offer advantages in terms of stability, physical properties, and absence of contaminants but also enable greater waste integration and require minimal production modifications, making them a highly advantageous option compared to other types of ceramics.

## 4. Conclusions

The partial conclusions drawn from the tests conducted in accordance with the standards affirm the ultimate objective of this research, which is to develop ceramic materials for bricks utilising 100% waste, specifically aggregate washing sludges and manganese mining residues. Thus, the partial conclusions are as follows:

- The washed aggregate sludges exhibit physical properties and chemical composition closely comparable to those of the clays traditionally employed in the manufacture of ceramic bricks, thus rendering this residue a viable substitute.
- Manganese mining residues have a higher density than both clays and washing sludges. However, their chemical composition does not reveal the presence of potentially harmful elements.
- In relation to mass loss, it can be observed that it does not vary too much, while, in linear shrinkage, an excellent quality of the materials obtained is observed, and even in percentages of 75% and 100% of Mn residue, there is a slight dilation of the shaped specimens.
- In the capillary water absorption test (suction), the results obtained show that the durability of the specimens is not affected.
- The cold and boiling water absorption tests show similar data, with absorption increasing as the percentage of Mn residue increases.
- In the results obtained for bulk density and open porosity, it is observed that the higher the amount of Mn residue, the higher the value obtained for these parameters.
- It is important to highlight that all the families of shaped specimens comply with the limitations in relation to compressive strength, even in the most unfavourable cases, which is the family made up entirely of Mn residues, from which a strength of 23.75 Mpa is obtained, which is higher than the 10 Mpa indicated in the standard.
- Some of the elements that have been vitrified in the ceramic material, thus reducing its impact on the environment, are silicon, manganese, aluminium, iron, and sodium, among others. This is an important aspect given that these elements are harmful to the environment and living beings.

As a final conclusion, it can be stated that the objectives of this study have been fully achieved. A ceramic material with acceptable mechanical performance and notable chromatic variety has been developed. However, the most significant aspect is its impact on sustainability and the circular economy. By utilising industrial by-products rather than virgin raw materials, this work not only significantly reduces environmental impact but also promotes circular economy principles by closing the loop on industrial waste. The developed process is not only environmentally friendly but also industrially scalable, allowing for integration into large-scale production. This approach contributes to more sustainable manufacturing practices in ceramics, advancing a more efficient and eco-friendly production model.

## Figures and Tables

**Table 1:** Composition of the different families of ceramic specimens formed with aggregate wash sludge and manganese residues.

Sample	% Sludge	% Mn Waste	% Water
M0	100	0	10
M25	75	25	10
M50	50	50	10
M75	25	75	10
M100	0	100	10

**Table 4:** X-ray fluorescence test results of aggregate washing sludge and manganese residues.

Element	Sludge (wt%)	Manganese Waste (wt%)
Si	32.45 ± 0.16	29.09 ± 0.17
Al	8.40 ± 0.08	1.29 ± 0.03
K	4.58 ± 0.15	0.228 ± 0.018
Na	1.97 ± 0.13	0.366 ± 0.041
Fe	1.70 ± 0.08	6.97 ± 0.15
Ca	0.417 ± 0.030	1.20 ± 0.07
Px	0.2020 ± 0.0083	0.1010 ± 0.0059
Mg	0.265 ± 0.029	0.292 ± 0.032
Ti	0.136 ± 0.002	0.0372 ± 0.0013
W	0.139 ± 0.007	-
Mn	-	12.41 ± 0.07

**Table 5:** Colourimetry test results.

Sample	Red	Green	Blue
M0	576.67	299.33	185
M25	205.67	150.33	115
M50	135	109.67	91.33
M75	107.33	89	76.33
M100	92	79.67	71.33
MC	315.33	164	114.33

## Data Availability

Data is contained within the article.
